# Chemopreventive effects of pterostilbene through p53 and cell cycle in mouse lung of squamous cell carcinoma model

**DOI:** 10.1038/s41598-021-94508-7

**Published:** 2021-07-21

**Authors:** Omchit Surien, Ahmad Rohi Ghazali, Siti Fathiah Masre

**Affiliations:** Programme of Biomedical Science, Center for Toxicology and Health Risk Studies (CORE), Faculty of Health Sciences, Universiti Kebangsaan Malaysia (UKM), Kuala Lumpur, Malaysia

**Keywords:** Cancer models, Cancer prevention

## Abstract

Cell proliferation and cell death abnormalities are strongly linked to the development of cancer, including lung cancer. The purpose of this study was to investigate the effect of pterostilbene on cell proliferation and cell death via cell cycle arrest during the transition from G1 to S phase and the p53 pathway. A total of 24 female Balb/C mice were randomly categorized into four groups (n = 6): N-nitroso-tris-chloroethyl urea (NTCU) induced SCC of the lungs, vehicle control, low dose of 10 mg/kg PS + NTCU (PS10), and high dose of 50 mg/kg PS + NTCU (PS50). At week 26, all lungs were harvested for immunohistochemistry and Western blotting analysis. Ki-67 expression is significantly lower, while caspase-3 expression is significantly higher in PS10 and PS50 as compared to the NTCU (*p* < 0.05). There was a significant decrease in cyclin D1 and cyclin E2 protein expression in PS10 and PS50 when compared to the NTCU (*p* < 0.05). PS50 significantly increased p53, p21, and p27 protein expression when compared to NTCU (*p* < 0.05). Pterostilbene is a potential chemoprevention agent for lung SCC as it has the ability to upregulate the p53/p21 pathway, causing cell cycle arrest.

## Introduction

Cancer is the second leading cause of death, responsible for 1 in every 6 deaths globally. Lung cancer is one of the most diagnosed types of human cancer and the leading cause of cancer deaths that killed 1.76 million people^1^. The high mortality rate among lung cancer patients is due to late diagnosis with advanced cancer stages, which resulted in the failure of standard treatment options such as chemotherapy^2, 3^. Lung cancer is classified into two types based on histology: non-small cell lung cancers (NSCLCs) and small cell lung cancers (SCLCs). NSCLCs are classified into several subtypes, including lung adenocarcinoma (AD), squamous cell carcinoma (SCC), and large cell carcinoma (LCC). NSCLCs are the most common type of lung cancer, accounting for nearly 80–85% of all cases^4–6^. Tobacco smoking has been shown to be a primary risk factor for lung cancer, but lung SCC carcinogenesis has the strongest link to tobacco smoking^7, 8^. Smoking cessation and tobacco prevention have been shown to effectively reduce the mortality rate among lung cancer patients^9^. Unfortunately, smoking cessation intervention among smokers to reduce the risk of lung cancer does not eliminate the significant risk, and the risk remains higher than among never smokers^10, 11^. As a result, there is a vital need to find effective chemoprevention agents to complement smoking cessation as an effective lung cancer prevention strategy. Pterostilbene is a natural analog to the well-studied compound resveratrol that has grown in popularity due to its metabolic stability and superior pharmacological activities^12, 13^. Pterostilbene was isolated for the first time from red sandalwood (*Pterocarpus santalinus*) and can be found mainly in a few natural sources, including grapes, blueberries, and *Pterocarpus marsupium*^14, 15^. Pterostilbene has been shown to have a few pharmacological properties that could lead to its development as an anticancer or chemoprevention agent, such as anti-inflammatory, antioxidant, anti-angiogenesis, and anti-proliferation properties^16–18^.

The most common mutated gene in many cancers, including lung cancer, is the *TP53* gene mutation, which encodes pivotal p53, a tumor suppressor protein^19, 20^. According to the Cancer Genome Atlas project, the most common mutated gene in lung SCC tumors is *TP53*, which can be found in more than 80% of lung SCC cases^21^. The p53 protein performs numerous functions, including DNA repair, cell cycle arrest, apoptosis induction, and cell senescence, in order to prevent or suppress tumor formation in response to stress responses such as DNA damage, hypoxia, hyperproliferation, and oncogenes activation^22, 23^. Tumor growth progression has been linked to an imbalance between cell proliferation and apoptosis, characterized by increased cell proliferation and/or decreased apoptosis^24, 25^. Ki-67 is the cell proliferation marker that has been widely used for decades to monitor tumor growth^26^. Ki-67 expression occurs throughout the active phases of the cell cycle of proliferating cells, beginning in the middle or late G1 phase, S, or G2 phase, and peaks during the M phase, but there is no expression of Ki-67 during the quiescent (G0) cells^27, 28^. Ki-67 overexpression in NSCLC patients is associated with a poor prognosis and a shorter survival time^29^. Cleaved caspase-3 is an important marker for apoptosis-induced cell death as caspase-3 is required for DNA fragmentation and cell morphological changes such as blebbing and shrinkage during apoptosis^30, 31^.

In response to a stress stimulus that can lead to cancer development, such as DNA damage, p53 will activate the downstream effectors of the protein p21, causing the cell cycle arrest^32, 33^. The protein p21 acts as a cyclin-dependent kinase (CDK) inhibitor, resulting cell cycle arrest by binding to CDK-1, CDK-2, and CDK 4/6^34^. The progression of the cell cycle from one phase to the next is driven and regulated by cyclins, which activate the catalytic enzyme of CDKs by binding to form cyclin-CDK complexes^35, 36^. In mammals, there are over 20 different types of cyclins and CDKs. Nonetheless, only a few are known to be involved as regulators in cell cycle progression, with various types of cyclin-CDKs complexes engaged during the various phases of G1, S, G2, and M to complete the cell cycle^37, 38^. Cyclin D and cyclin E, for example, play critical roles in inactivating CDKs during the cell cycle transitions from G1 to S^39, 40^.

## Results

### Pterostilbene reduced cell proliferation while increasing apoptosis in the bronchial epithelium layer

**Figure 1:**
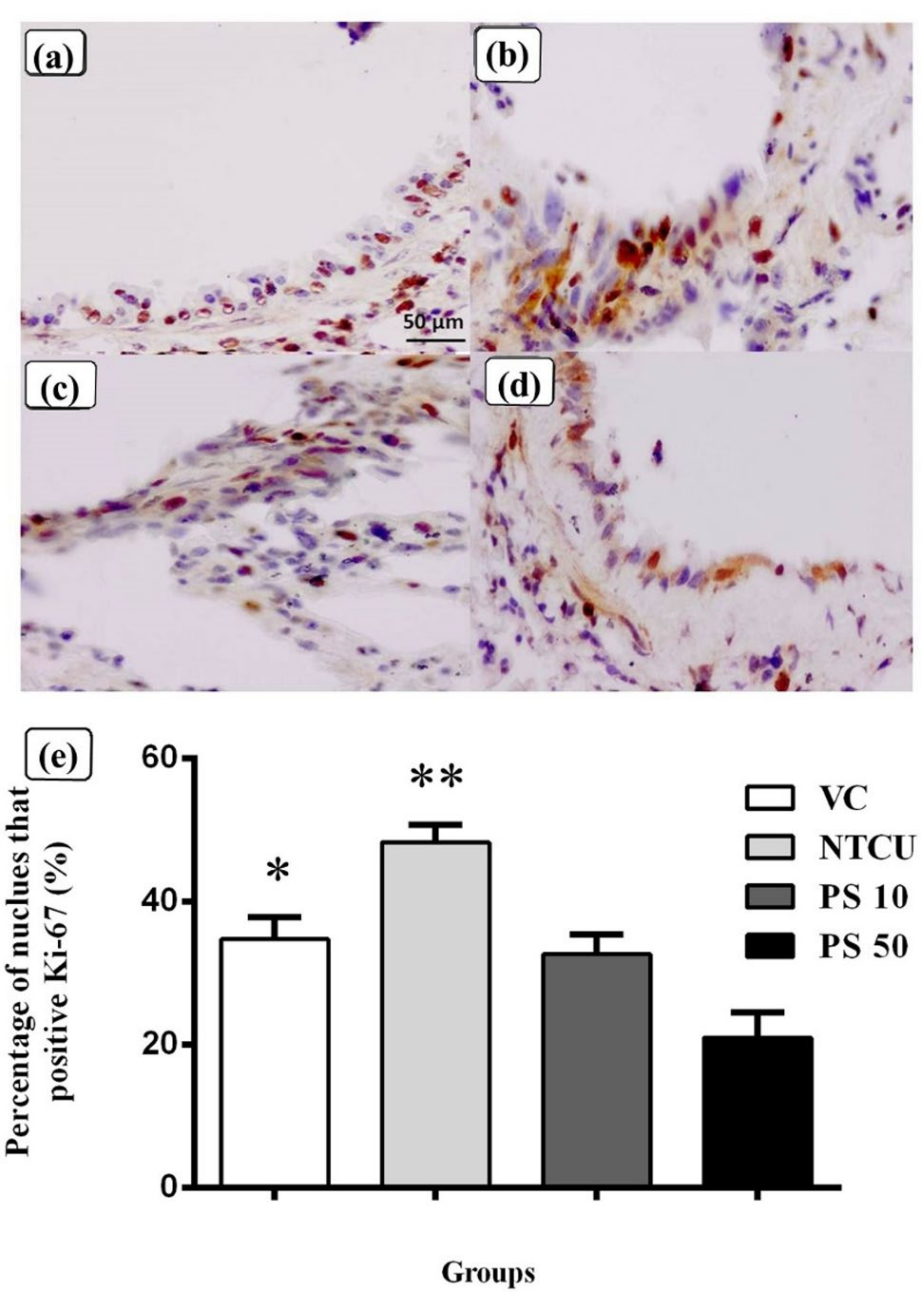
Immunohistochemistry was performed to detect the cell proliferation marker Ki-67 in paraffin-embedded lung tissues. (**a**) Showed the bronchial epithelium layer of a mouse from the VC control group, which contained fewer immunopositive cells (brown-staining nucleus). (**b**) of the NTCU group that was treated with NTCU showed the highest level of Ki-67 expression with abundant immunopositive cells at the bronchial epithelium layer when compared to other groups. The two pterostilbene treatment groups, (**c**): PS 10 and (**d**): PS 50 showed a decrease in Ki-67 expression with fewer immunopositive cells at the bronchial epithelium layer compared to (**b**) of the NTCU group. (**e**) showed the quantification of cell proliferation activities using the percentage of nucleus that was positive for Ki-67 (immunopositive cells) per total number of nuclei at the bronchial epithelium layer. Data are presented as mean ± SEM. *Statistically significant differences between VC and NTCU and PS 50 (*p* < 0.05). **Statistically significant differences between NTCU and PS 10 and PS 50 (*p* < 0.05).

The immunohistochemistry of Ki-67 protein on the formalin-fixed paraffin-embedded lung sections was used to determine cell proliferation activity. Figure 1 shows the bronchial epithelium layer of a representative mouse from the control and pterostilbene groups. Figure 1a depicted the immunopositive bronchial epithelium layer (brown-staining nucleus) of the VC mouse. Figure 1b shows that the NTCU group had the highest level of Ki-67 expression compared to other groups with numerous immunopositive cells in the bronchial epithelium layer. Figure 1c,d show a decrease in Ki-67 expression levels in both PS 10 and PS 50 when compared to NTCU. Figure 1e depicted the percentage of nucleus that is positive for Ki-67 staining per total number of nuclei in the bronchial epithelium layer. The NTCU group had a significantly higher percentage of Ki-67 positive nuclei (48.30 ± 2.37%) than the VC group (34.60 ± 2.96%) (*p* < 0.05). Pterostilbene treatment reduced cell proliferation activities, with PS 10 (32.61 ± 2.74%) and PS 50 (20.98 ± 3.51%) significantly lower than in the NTCU group (*p* < 0.05).

**Figure 2:**
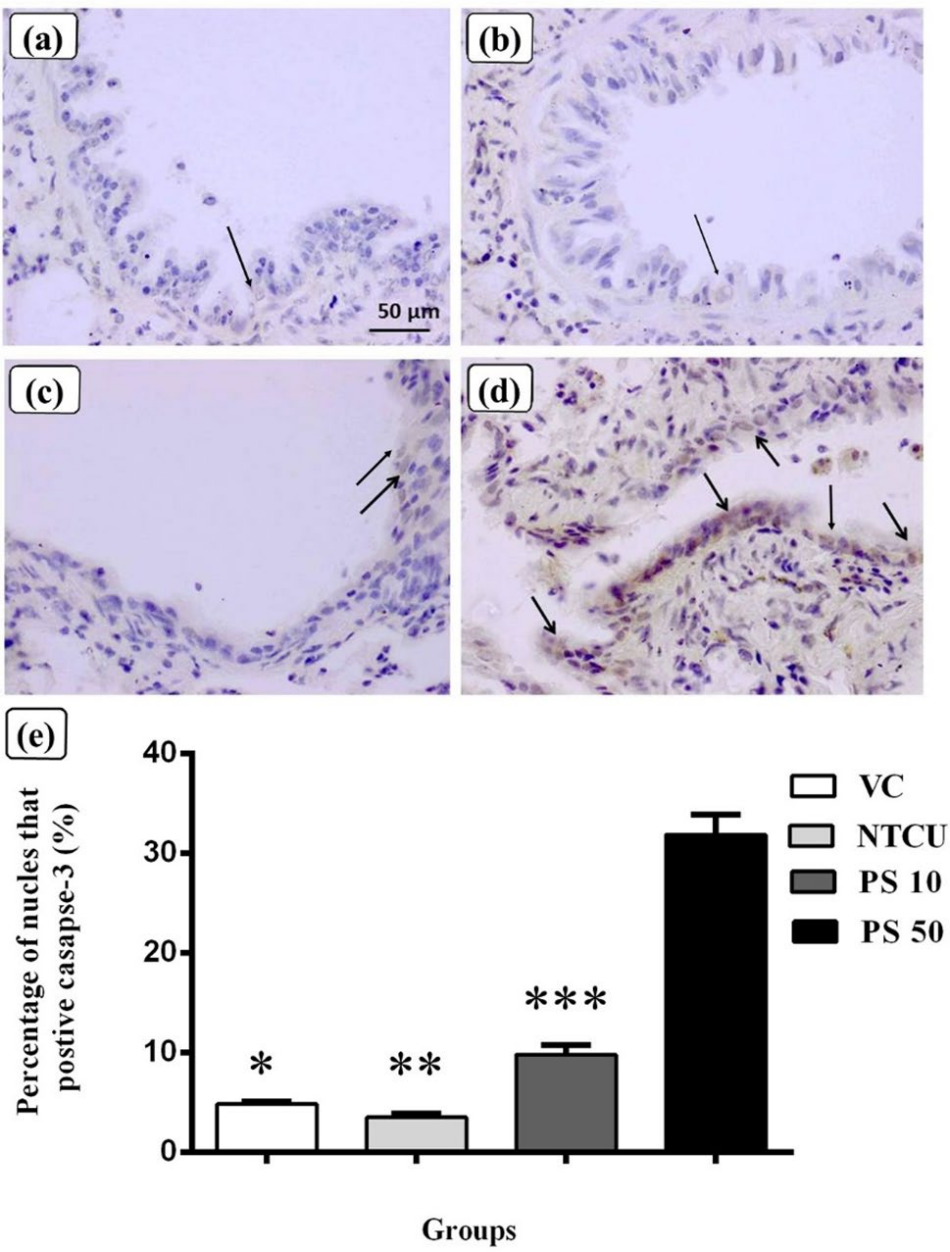
Immunohistochemistry was performed to detect the apoptosis marker cleaved caspase-3 in paraffin-embedded sections. Figure showed the bronchial epithelium layer of a representative mouse from each group, as shown in (**a**): VC. (**b**) shows an NTCU with fewer caspase-3 immunopositive epithelial cells (arrow). (**c**): PS 10 had an increase in caspase-3 immunopositive epithelial cells (arrows), and (**d**): PS 50 had a lot of caspase-3 immunopositive epithelial cells (arrows). (**e**) Showed the quantification of apoptosis activities using the percentage of nucleus positive for caspase-3 (immunopositive cells) per total number of nuclei at the bronchial epithelium layer. Data are presented as mean ± SEM. *there is a statistically significant between VC compared to PS 50 (*p* < 0.05). **Statistically significant differences between NTCU and PS 10 and PS 50(*p* < 0.05). ***Statistically significant difference between PS 10 and PS 50 (*p* < 0.05).

The immunohistochemistry of cleaved caspase-3 was performed on formalin-fixed paraffin-embedded lungs to investigate the effect of pterostilbene on the apoptosis of bronchial epithelial cells in the lungs. Figure 2 depicts cleaved caspase-3 protein expression in the bronchial epithelium layer in the control and pterostilbene treatment groups. Figure 2a,b show fewer immunopositive (brown-staining nucleus) epithelial cells in the VC and NTCU groups, respectively. In contrast, the pterostilbene treatment groups in Fig. 2c: PS 10 and Fig. 2d: PS 50 had higher levels of cleaved caspase-3 expression than the VC and NTCU. When compared to the other groups, the PS 50 groups had the highest level of cleaved caspase-3 expression, with numerous immunopositive epithelial cells in the bronchial epithelium layer. Figure 2e showed apoptosis activity in the bronchial epithelium layer as a percentage of cleaved caspase-3 positive nucleus per total number of nuclei. The NTCU group had the lowest rate (3.49 ± 0.37%) when compared to the other groups. The VC group outperformed the NTCU with 4.81 ± 0.25%; however, there is no significant difference between the VC and the NTCU (*p* > 0.05). The results of the pterostilbene treatment groups revealed that the expression levels of cleaved caspase-3 were significantly higher in PS 10 (9.78 ± 0.97%) and PS 50 (31.84 ± 2.03%) groups than in the NTCU group (*p* < 0.05).

### Pterostilbene upregulated p53 and its downstream effector protein p21

**Figure 3:**
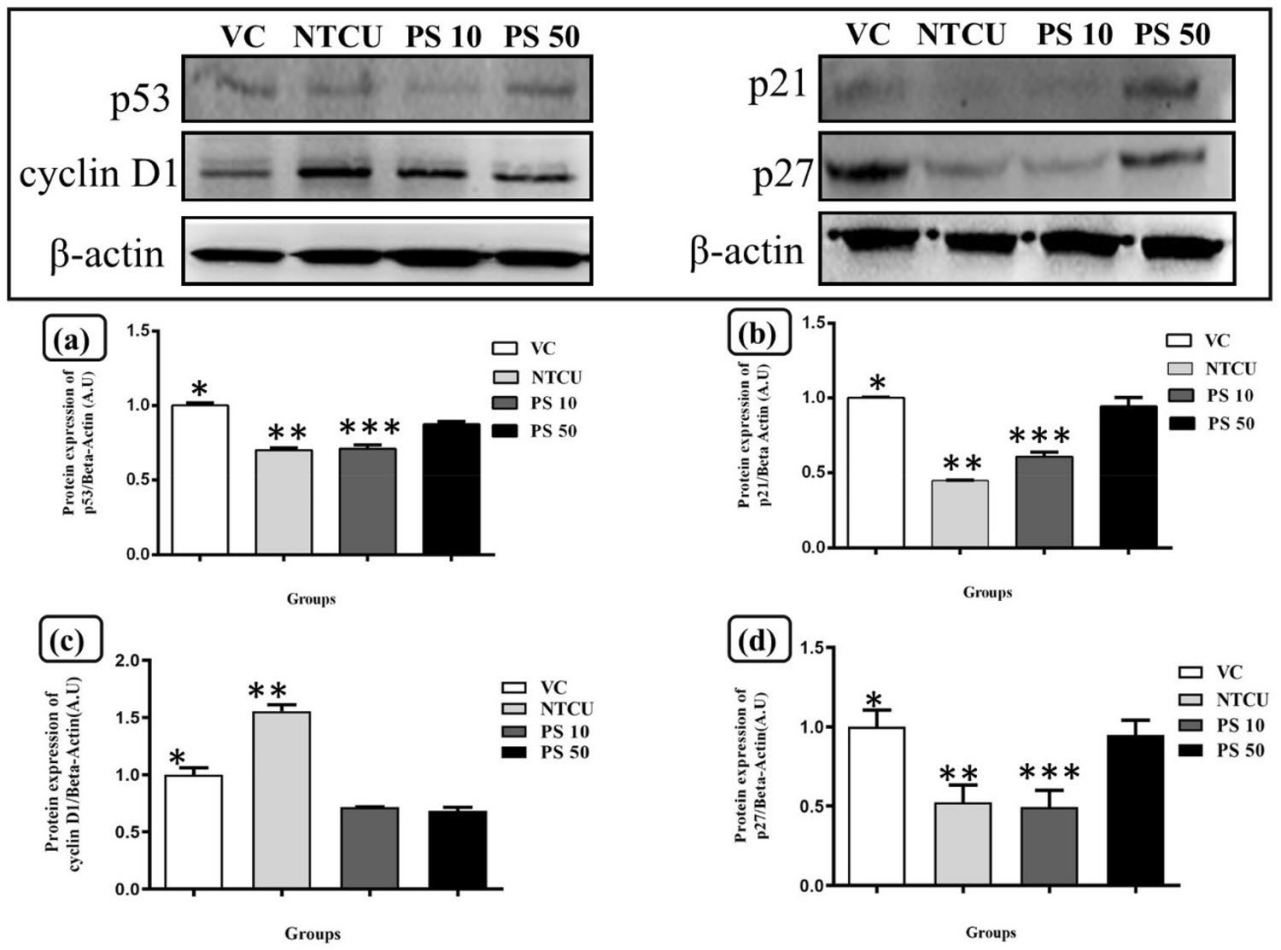
Western blot of protein expression of p53, p21, p27 and cyclin D1 in mouse lung tissue with the representative images of protein bands for each group with VC: vehicles control group; NTCU: cancer control group treated with NTCU; PS 10: 10 mg/kg of pterostilbene treatment with NTCU, and PS 50: 50 mg/kg of pterostilbene treatment with NTCU. (**a**) Showed the quantification graph of the arbitrary unit, A.U of p53 expression. *Statistically significant differences between VC and NTCU, PS 10 and, PS 50 (*p* < 0.05). **Statistically significant difference between NTCU and PS 50 (*p* < 0.05). *** Statistically significant difference between PS 10 and PS 50 (*p* < 0.05). (**b**) Showed the quantification graph of the arbitrary unit, A.U of p21 expression. *Sstatistically significant difference between VC and NTCU (*p* < 0.05). **Statistically significant difference between NTCU and PS 50 (*p* < 0.05). ***Statistically significant difference between PS 10 and PS 50 (*p* < 0.05). (**c**) Showed the quantification graph of the arbitrary unit, A.U of cyclin D1 expression. *There are statistically significant differences between VC and NTCU and PS 10 (*p* < 0.05). **Statistically significant difference between NTCU and PS 50 (*p* < 0.05). ***Statistically significant difference between PS 10 and PS 50 (*p* < 0.05). (**d**) Showed the quantification graph of the arbitrary unit, A.U of p27 expression. *Statistically significant differences between VC and NTCU and PS 10 (*p* < 0.05). **Statistically significant difference between NTCU and PS 50 (*p* < 0.05). ***Statistically significant difference between PS 10 and PS 50 (*p* < 0.05).

As shown in Fig. 3, Western blot analysis was used to investigate the effect of pterostilbene treatment on the expression of p53, p21, p27, and cyclin D1. The p53 western blot quantification graph in Fig. 3a was normalized with the housekeeping protein beta-actin. When compared to the NTCU, the PS 50 group significantly increased p53 protein expression (*p* < 0.05). However, no significant differences in p53 expression were found between the PS 10 and NTCU groups (*p* > 0.05). Figure 3b depicted a graph of p21 protein expression levels to investigate the p53/p21 pathway cell cycle arrest. This study found a significant decrease in p21 expression in the NTCU group compared to the VC group (*p* < 0.05), which was similar to the results for p53 protein expression. When the high dose pterostilbene treatment group of PS 50 was compared to the NTCU treatment group, the PS 50 significantly increased the p21 expression (*p* < 0.05). The low dose of pterostilbene in the PS 10 group increased p21 protein expression compared to the NTCU group; however, there was no significant difference in the level of p21 expression between PS 10 and NTCU groups (*p* > 0.05). These findings show that a high dose of pterostilbene (PS 50) treatment significantly increased the expression of p53 and its downstream effector p21 when compared to NTCU.

### Pterostilbene induced cell cycle arrest by upregulating p27 and downregulating the cell cycle regulatory proteins, cyclin D1 and E2

Figure 3 showed the Western Blot results of the cell cycle markers cyclin D1 and p27, along with protein band images and a quantification graph. Figure 3c depicted a quantification graph of cyclin D1 protein expression normalized with beta-actin. Overexpressed cyclin D1 was found in the NTCU group, where there was a significant increase in cyclin D1 expression compared to the VC group (*p* < 0.05). When compared to the NTCU group, pterostilbene treatment significantly reduced cyclin D1 expression in PS 10 and PS 50 (*p* < 0.05). Figure 3d showed a graph of p27 expression quantification normalized with beta-actin as the house-keeping protein. The expression level of p27, a cell cycle inhibitor, was significantly lower in the NTCU group compared to the VC group (*p* < 0.05). The low dose pterostilbene treatment group of PS 10 showed no significant difference in p27 protein expression when compared to the NTCU group (*p* > 0.05). However, when compared to the NTCU, the PS 50 group significantly increased p27 expression (*p* < 0.05).

**Figure 4:**
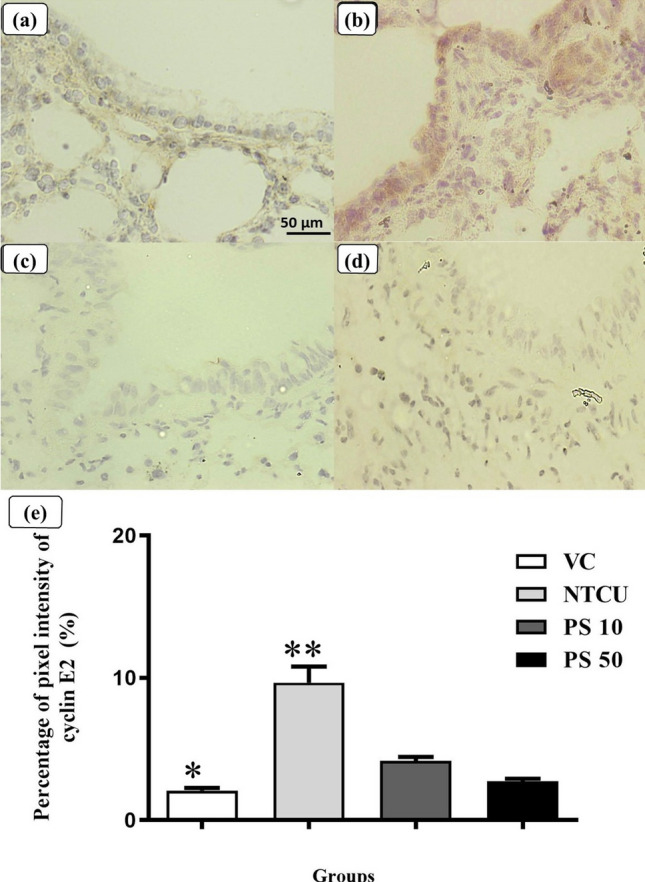
Immunochemistry staining of cyclin E2 cell cycle marker for each group focused on the bronchial epithelium layer of the lung. The immunopositive area of cyclin E2 is represented by the brown staining. (**a**): Low cyclin E2 expression in the VC group. (**b**): Strong nuclear and cytoplasmic cyclin E2 expression at the bronchial epithelium layer in the NTCU group. (**c**): PS 10, and (**d**): PS 50 depict very low expression of cyclin E2 at the bronchial epithelium layer. (**e**) showed the quantification of cyclin E2 as a percentage of pixel intensity (%) using the Fiji/Image J software. *Statistically significant differences between VC and NTCU, PS 10, and PS 50 (*p* < 0.05). **Statistically significant differences between NTCU and VC, PS 10, and PS 50 (*p* < 0.05).

Figure 4 depicts the immunohistochemistry staining of cyclin E2 in the bronchial epithelium for each group. In Fig. 4a, the representative mouse of the control group, VC, showed a very weak brown-color staining of cyclin E2 in the bronchial epithelium layer. In contrast, the NTCU group had a high level of cyclin E2 expression, with a large area of intense brown staining observed in the bronchial epithelium layer, as shown in Fig. 4b. The pterostilbene groups of PS 10 in Fig. 4c and PS 50 in Fig. 4d had lower cyclin E2 expression than the NTCU group, with a low brown staining bronchial epithelium layer that resembled the staining pattern in the VC group.

## Discussion

Cancer chemoprevention has grown in popularity, particularly from natural compounds found in various natural sources that have the potential to postpone, delay, or stop carcinogenesis. Pterostilbene is a natural compound that has been demonstrated to inhibit various types of human cancer cell lines (in vitro) and to prevent tumor formation induced by various carcinogens in animal models of in vivo studies. The molecular events of pterostilbene in the prevention of NTCU-induced lung SCC animal models, on the other hand, have not been studied. Based on histopathological findings, we reported that pterostilbene pre-treatment (chemopreventive effects) prevented the development of lung SCC in the mouse model^41^. Previously, we demonstrated that a low dose of pterostilbene (10 mg/kg) could prevent carcinogenesis at the pre-malignant stage of hyperplasia. In contrast, a high dose of pterostilbene (50 mg/kg) preserved the normal histology of the lungs with single ciliated columnar epithelium. In contrast, mice treated with NTCU without pterostilbene developed lung SCC with invasion of the basement membrane and disorganization of epithelial cells with an increased nucleus:cytoplasm (N:C) ratio^41^. As a results, this study was carried out to determine the mechanisms responsible for pterostilbene to act as a chemopreventive agent against lung SCC carcinogenesis in the mouse model.

Carcinogenesis has been linked to an imbalance between cell proliferation and cell death, with carcinogenesis defined by an increase in cell proliferation and a decrease in cell death processes such as apoptosis^42, 43^. Protein Ki-67 is a proliferation marker that is closely associated with cell proliferation during the active phases of the cell cycle (G1, S, G2, and mitosis), and it is absent in resting cells during the G0 phase. Ki-67 expression is commonly used as a proliferation marker to monitor tumor development, progression, and aggressiveness^44, 45^. Ki-67 expression in NSCLCs tumors has been linked to a poor prognosis in NSCLC patients when compared to NSCLC patients with low Ki-67 expression^28, 46^. Previous studies have shown that the NTCU induced lung SCC mouse model causes cellular hyper-proliferation, as evidenced by an increase in Ki-67 expression^47, 48^. In our study, pterostilbene treatment groups demonstrated a decrease in Ki-67 expression and thus decreased cell proliferation when compared to the NTCU group to prevent the growth of lung SCC, as an increase in cell proliferation is associated with an increased risk of carcinogenesis^49^.

Moreover, the anti-proliferative effects of pterostilbene via Ki-67 down-regulation have been demonstrated by immunohistochemistry staining on other types of cancers via in vivo studies such as skin melanoma, prostate cancer, and hepatocellular carcinoma^18, 50, 51^. Aside from anti-proliferative effects, this study found that pterostilbene can increase apoptosis by increasing the expression of cleaved or activated caspase-3. This finding is similar to previous studies on lung adenocarcinoma, colon, and esophageal cancers in which pterostilbene was found to increase cleaved caspase-3 activity^52–54^. Furthermore, a previous in vitro study of diffuse large B-cell lymphoma cells revealed that pterostilbene inhibited cell growth by increasing caspase-3 activity^55^. Caspase-3 is a reliable apoptosis marker that also acts as a crucial apoptosis mediator. Caspase-3 participates in DNA fragmentation, morphological changes (formation of the apoptotic body), and biochemical events during apoptosis^31, 56^. In addition to caspase-3 expression for apoptosis and Ki-67 expression for cell proliferation analysis, the TUNEL assay and terminal deoxynucleotidyl transferase(TdT) dUTP Nick-End Labelling (TUNEL) assay and bromodeoxyuridine (BrdU) staining can be considered in the future to support the effects of pterostilbene on apoptosis and cell proliferation^57, 58^.

In response to the hyper-proliferation and decreased apoptosis activities during carcinogenesis, p53 plays a vital role in transactivating various targeted genes to cause cell death via apoptosis and/or cell cycle arrest^59, 60^. The effect of pterostilbene on p53 upregulation has been reported in the hepatocellular mouse carcinoma model, which is consistent with our discovery that pterostilbene can upregulate p53^61^. The *TP53* gene mutation, which encodes p53, is one of the most common mutations in NSCLCs, with the *TP53* gene mutation occuring more frequently in lung SCC than in lung AD. The *TP53* mutation was found in approximately 51% of lung SCC cases, which is higher than the 36% cases found in lung AD^62, 63^. Some stressful events, such as DNA damage and oncogene activation, can lead or stimulate the development of cancer. In response to that cancer stimulus, p53 is activated and contributes to the upregulation of its downstream effector, p21 and p21 acts as a cell cycle inhibitor^64^. Aside from our findings, pterostilbene has been shown to have anticancer effects on different human lung cancer cell lines via a p53 dependent pathway. For example, pterostilbene has been shown to cause cell senescence and inhibition on human A549 lung adenocarcinoma cells and human H460 large-cell lung carcinoma cells via p53 upregulation, which is similar to our findings^65, 66^. These two previous studies, however, differ from ours in that they reported the effect of pterostilbene on p53 expression in lung cancers other than lung SCC. Moreover, in vitro assyas on lung cancer cell lines revealed that pterostilbene upregulated p53 expression. As a result, our study is among the first to demonstrate that pre-treatment with pterostilbene increased p53 expression in NTCU induced lung SCC mouse model (in vivo).

Moreover, we found that pterostilbene treatment induced p53 upregulation, which coincided with p21 upregulation. Furthermore, Pan et al. (2007) showed that pterostilbene could upregulate p53 and p21 to induce cell cycle arrest at the G1 phase in human gastric carcinoma cells^67^. p21 is a cyclin-dependent kinase (CDK) inhibitor that can bind to and inhibit the role of CDK-1, CDK-2, and CDK-4 in causing cell cycle arrest during the transition from G1 phase to S phase^32, 68^^.^ Furthermore, several studies have found a link between p21 expression and the prognosis of lung SCC patients. Patients with lung SCC who had high p21 expression had a significantly higher survival rate than patients with low p21 expression^69, 70^. Aside from p21, another CDK inhibitor known as p27 is involved in cell cycle control during the G1 phase^71^. In normal cells, the p27 level is high during the G0 and early G1 phases, but it decreases during the G1 phase to allow cell cycle progression to the next phase. Thus, overexpression of p27 is necessary to induce cell cycle arrest in the G1 phase before the cell enters the S phase^72, 73^. An increase in p27 expression may cause cell death via apoptosis in addition to causing cell cycle arrest^74, 75^. We discovered that pterostilbene treatment increased the expression of p27 and cleaved caspase-3 (apoptosis), which was consistent with the findings of an in vitro lung SCC cell line (H20) study in which pterostilbene increased the activity of apoptosis activity along with p27 expression^55^.

A cell cycle is a four-phase event that occurs during the production of new cells. The event begins with the G0/G1 phase (a static phase in which the cell prepares to enter the S phase with protein and organelle sysnthesis); the S-phase (during which DNA replication occurs); the G2 phase (a static phase during which the cell prepares to enter the M phase); and finally the M-phase (a division of the nucleus and followed by a cytoplasmic division)^76, 77^. The cyclin-CDK complexes regulate the progression of the cell cycle from one phase to the next. The CDKs must bind to the cyclins in order to become active and facilitate cell cycle progression^78^. CDK inhibitors like p21 and p27 cause cell cycle arrest by binding to and inhibiting the catalytic function of the cyclin-CDK complex^39, 79^. Progress through each phase of the cell cycle is mediated by a specific cyclin-CDK complex, with cyclin D-CDK4/6 and the cyclin E-CDK2 mediating the transition from G1 to S^38^. Cell cycle arrest in the early phase of the cell cycle is crucial to cancer prevention intervention and its effectiveness, as cell cycle arrest in the G1 phase before entering the S phase of the cell cycle prevents the replication of damaged DNA, which could lead to carcinogenesis, and allows a cell to repair the DNA damage^80, 81^.

In this study, pterostilbene treatment caused a cell cycle arrest during the transition from G1 to S phase, as well as a decrease in cyclin D1 and E2 expression. There are a few different types of cyclin-CDK complexes that control the checkpoints in the cell cycle during the transitions between different phases in the completion of cell cycle, and each checkpoint is controlled by a different cyclin-CDK complex. For example, the transition from the G1 to S phase of the cell cycle is highly regulated by the complexes of cyclin D and E with CDKs^82^. There are only a few types of cyclin D, such as cyclin D1 and D2^83^, but abnormal cyclin D1 expression is more common in lung SCC tumors, with 68% of lung SCC tumors having cyclin D1 overexpression^84^. More importantly, our discovery of cyclin D1 downregulation by pterostilbene was supported by another in vivo study that revealed pterostilbene treatment could reduce cyclin D1 expression to reduce colon tumor multiplicity in rats^85^. The downregulation of cyclin E2 by pterostilbene in this study also strengthens the ability of pterostilbene to induce cell cycle arrest at the G1 to S phase transition, as cyclin E2 overexpression is associated with a shortening of the G1 phase duration and the catalytic activity of cyclin E2 is maximal during the G1 to S phase transition of cell cycle progression^86, 87^.

Despite the fact that many previous studies have been conducted to investigate the effects of pterostilbene on lung SCC, our study focuses on the chemopreventive effects of pterostilbene on healthy lungs of the mouse model (in vivo) against chemically induced lung SCC. Schneider et al. (2010), for example, discovered that pterostilbene inhibits the human lung SCC cell line (SK-MES-1) in vitro^88^. In addition, pterostilbene has also been reported to have antitumor effects on lung SCC in vitro and in vivo (tumor xenograft model) assays on another human lung SCC cell line (H520 cells). Pterostilbene was found to be able to upregulate p21 and p27, and downregulate cyclin E and A in H20 cells (human lung SCC)^89^. In the same study, a 50 mg/kg intraperitoneal injection of pterostilbene on an H520 cells xenograft model (in vivo) in BALB/c mice significantly reduced tumor weight and volume^89^. This past finding^89^ has shown the potential of pterostilbene as a cell cycle inhibitor, which is in concordance to our results that reveal upregulation of p21 and p27, and downregulation of cyclin D1 and E2 in the pterostilbene treated groups. However, further investigation on the effect of pterostilbene on other cell cycle markers involved in lung cancer, such as cyclin A and B expression, is needed to support cell cycle arrest by pterostilbene^90^.

Pterostilbene treatment may also help to increase survival time in the animal model. Resveratrol, a natural analog of pterostilbene, has been shown to improve survival rate in a mouse xenograft model, in which mice were injected with B16F10 cells (melanoma) and had metastasis to the lungs. After a month, 70% of the resveratrol-treated mice survived with fewer metastatic colonies in the lungs, whereas all of the control mice had died^91^. In addition, mice treated with pterostilbene in xenograft tumors of human breast cancer cells (MDA-MB-231 cell lines) had a longer survival time. This is because Su et al. (2015) found that mice with xenograft tumors treated with pterostilbene had a longer survival time compared to the vehicle-treated control mice^92^. As a result, pterostilbene treatment has the potential to increase mouse survival time or rate because it delayed the lung SCC carcinogenesis induced by NTCU. However, in a future study, the experimental period should be extended to investigate the effect of pterostilbene treatment on mouse survival in an NTCU-induced lung SCC model.

## Conclusion

Pterostilbene inhibited lung SCC mouse carcinogenesis by inducing cell cycle arrest in the G1-S phase transition via the downregulation of cyclin D1 and E2 and the upregulation of p53 and its downstream effectors, p21, resulting in cell proliferation inhibition and apoptosis induction. Our findings suggest that pterostilbene may be a potential chemopreventive agent against lung SCC; hence, future research into epigenetic involvement may be conducted to support this study.

## Methodology

### Materials

The pterostilbene compound with a purity of 98% was obtained from Friedemann Schmidt Chemicals. The N-*nitroso-tri-chloroethylurea*, (NTCU) was purchased from Toronto Research Chemicals, Inc. The primary antibodies were purchased from different companies with cytokeratin 5/6 (Biobyrt United Kingdom, UK); Ki-67 (Merck, Germany); p21 and p27 Proteintech, United States); β-actin, caspase-3 and cyclin E2 and D1 (Cell Signalling Technology, United States).

### Animal model

All methods were carried out in accordance with relevant guidelines and regulations. All experimental protocols in this study were approved by the Universiti Kebangsaan Malaysia Animal Ethical Committee (UKMAEC: FSK/2017/FATHIAH/24-MAY/846-MAY-2017MAY-2020). This study was carried out in compliance with the ARRIVE guidelines. Balb/C mice were obtained at 5 weeks of age from the Faculty of Veterinary, Universiti Putra Malaysia (UPM). Mice were acclimatized for 2 weeks after arrival to help them adjust to their new surroundings. At 7 weeks of age, 24 Bablb/c mice were randomly divided into four groups. The first group was the vehicle control (VC), in which mice were treated topically with 70% acetone at the shaved dorsal subscapular area and intraperitoneally with corn oil. Mice in the negative control group (NTCU) were given corn oil and 25 µL of 0.04 M NTCU in 70% acetone. There are two pterostilbene treatment groups, each with a low and high pterostilbene dose. To investigate the chemopreventive effect of pterostilbene on lung SCC carcinogenesis induced by NTCU in mouse lungs, pterostilbene was given 2 weeks before the first NTCU exposure and continued throughout the experimental period with 30 min before each NTCU topical application. Mice in the low-dose pterostilbene group were given 10 mg/kg pterostilbene dissolved in corn oil and NTCU topically (PS 10 + NTCU). Mice in the high-dose pterostilbene group were given 50 mg/kg pterostilbene in corn oil as well as NTCU topical application until the end of the experiment (PS 50 + NTCU). After 26 weeks of twice-weekly pterostilbene and NTCU treatments, the mice were anesthetized with a ketamine/xylazine (KTX) cocktail and sacrificed via cervical dislocation. The lungs were extracted and divided in order to perform Western blot and immunohistochemistry tests.

#### Immunohistochemistry staining for caspase-3, Ki-67, and cyclin E2 protein expression

The lung tissues were fixed in 10% buffered formalin at 4 °C for 20 h (less than 24 h) before being transferred to 70% ethanol at 4 °C. For immunohistochemistry staining, paraffin-embedded tissues were sectioned at a thickness of 4 µm. Ki-67 (1: 100), cyclin E2 (1: 100), and caspase-3 (1:200), were the primary antibodies and dilution factors used for immunohistochemistry staining, which were diluted in 10% normal goat serum blocking solution. The tissue sections were washed with PBS on the second day before being incubated with horseradish peroxidase (HRP) (1: 100) secondary antibody diluted with 20% bovine serum albumin (BSA). The tissue slides were washed with PBS before being stained with a freshly prepared 3′3-diaminobenzidine tetrahydrochloride (DAB) solution and counterstained with hematoxylin. The Ki-67 and caspase-3 immunostaining results were reported as the percentage of immunoreactive cells for stained nucleus (brown staining indicates the presence of the target antigen or protein) over the total number of nuclei (stained and unstained nuclei) in the bronchial epithelium layer of the lungs^93^. FIJI/Image J (Java 8 version 64-bit) software was used to quantify cyclin E2 protein expression with nuclear and cytoplasmic immunohistochemistry staining, and the percentage of pixel intensity was reported^94^.

#### Protein expression of p53, p21, p27, and cyclin D1 analysis by western blotting

Lung tissues were homogenized with a mixture of RIPA buffer and protease inhibitor cocktails (Merck, USA) to lyse the cells. The homogenate was then centrifuged at 14 000 rpm for 25 min at 4 °C, and the supernatant was collected, aliquoted, and stored at − 20 °C in appendorf tubes. The BCA Protein Assay Kit (Merck, USA) was used to determine protein concentrations. The proteins were separated on a 12% polyacrylamide gel electrophoresis using 10 µg of each sample in a loading buffer for each well. Following electrophoresis, the proteins were transferred to a polyvinylidene difluoride (PVDF) membrane (Bioflow, Malaysia) and mashed with Tris Buffered Saline with Tween 20 (TBST). The membranes were then blocked in 5% BSA for 1 h at room temperature, washed with TBST, and incubated with a primary antibody diluted in 5% BSA overnight on the shaker at 4 °C. With a 1: 100 dilution factor, the primary antibodies used in this experiment were p53, p21, p21, and cyclin D1. The membranes were washed with TBST the next day before being incubated with horseradish peroxidase (HRP) conjugated secondary antibodies at room temperature for 1 h on a shaker. After washing the membranes with TBST. the immunoreactive bands on the membrane were detected and quantified using enhanced chemiluminescence.

#### Statistical analysis

All data were presented in the mean ± standard error mean (SEM). The data were tested for normality distribution by using the Shapiro–Wilk test with the SPSS version 25 software. The Shapiro–Wilk test was used as the sample size in this study is small with only 6 (n = 6), and data with *p* more than 0.05 (*p* > 0.05) is considered as normally distributed and vice versa. The One-Way analysis of variance (ANOVA) statistical test was used for the normally distributed data, and the Kruskal–Wallis one-way analysis of variance (ANOVA) was used for data that were not normally distributed. The *p* < 0.05 value was considered to be a statistically significant difference.

## Supplementary Information

Supplementary Information.
