# Supplementary material for: Chemopreventive effects of pterostilbene through p53 and cell cycle in mouse lung of squamous cell carcinoma model
Source: Sci Rep. 2021 Jul 21;11:14862. doi: 10.1038/s41598-021-94508-7 (PMC8295275; doi:10.1038/s41598-021-94508-7)
Supplement: Supplementary file 1 — Supplementary Information. [file 41598_2021_94508_MOESM1_ESM.docx]

**Supplementary Materials**


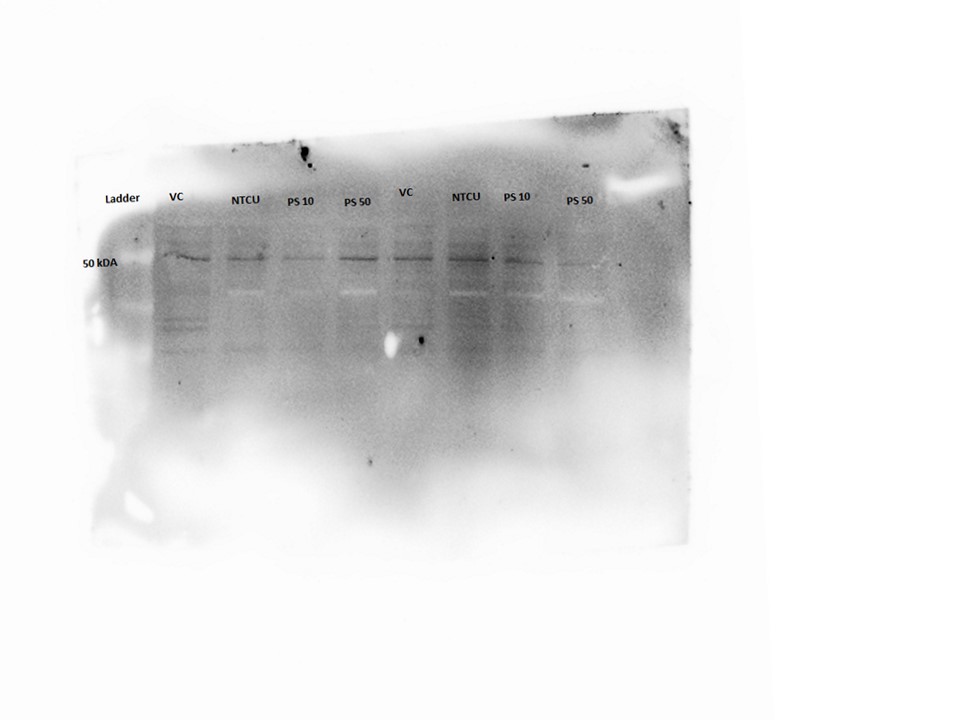


**Supplement Figure 1:** The full blot of the p53 protein band that is included in Figure 3 of the main manuscript. VC: vehicles control group; NTCU: cancer control group treated with NTCU; PS 10: 10 mg/kg of pterostilbene treatment with NTCU, and PS 50: 50 mg/kg of pterostilbene treatment with NTCU.


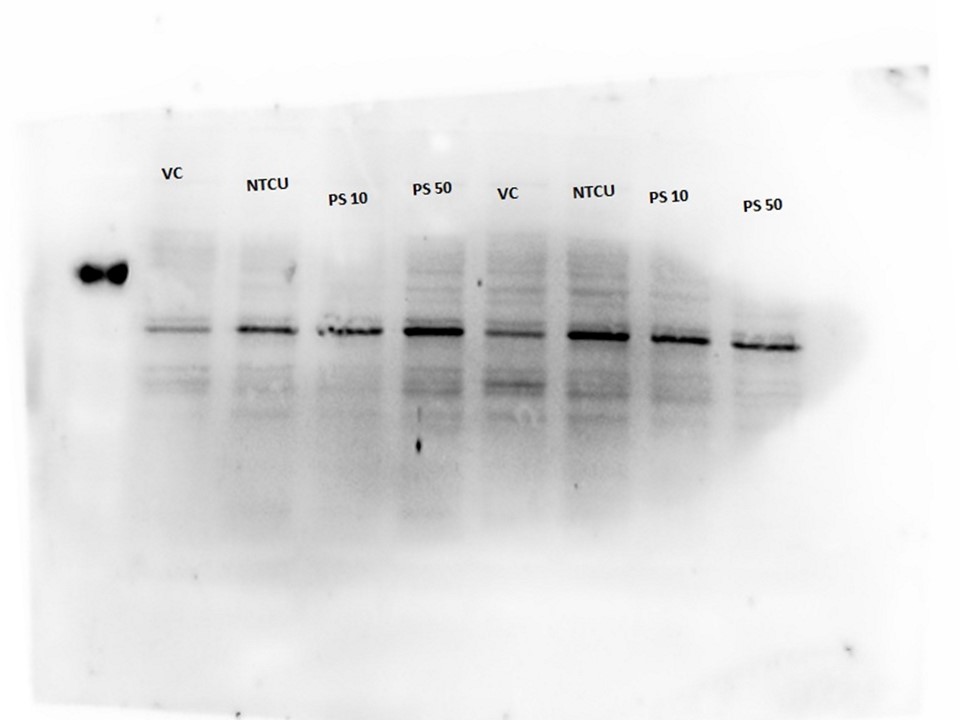


**Supplement Figure 2:** The full blot of the cyclin D1 protein band that is included in Figure 3 of the main manuscript. VC: vehicles control group; NTCU: cancer control group treated with NTCU; PS 10: 10 mg/kg of pterostilbene treatment with NTCU, and PS 50: 50 mg/kg of pterostilbene treatment with NTCU.


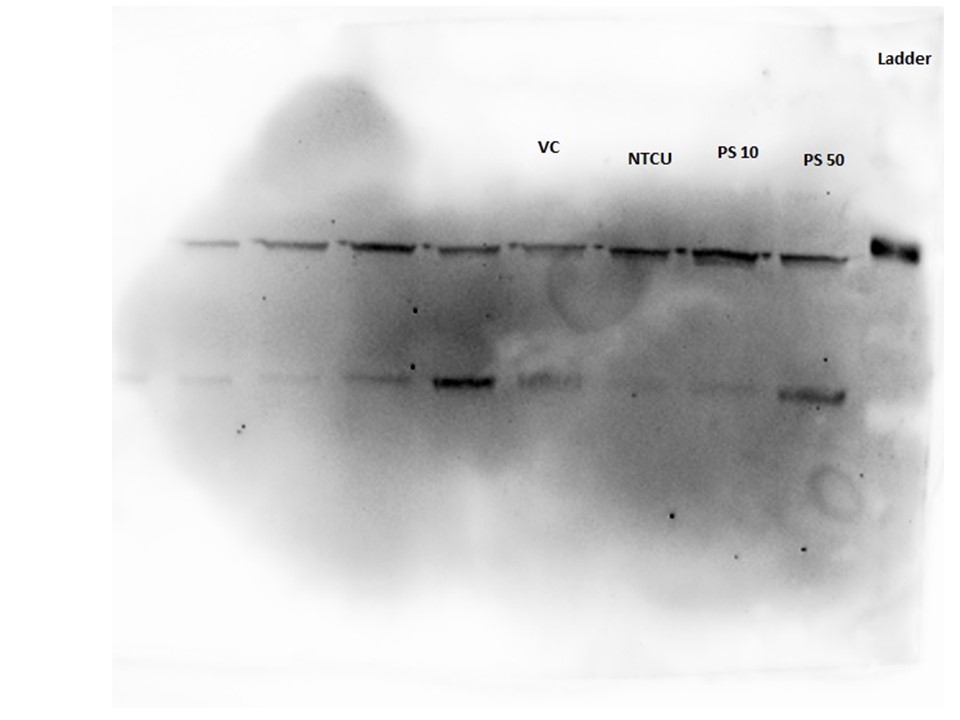


**Supplement Figure 3:** The full blot of the p21 protein band that is included in Figure 3 of the main manuscript. VC: vehicles control group; NTCU: cancer control group treated with NTCU; PS 10: 10 mg/kg of pterostilbene treatment with NTCU, and PS 50: 50 mg/kg of pterostilbene treatment with NTCU.


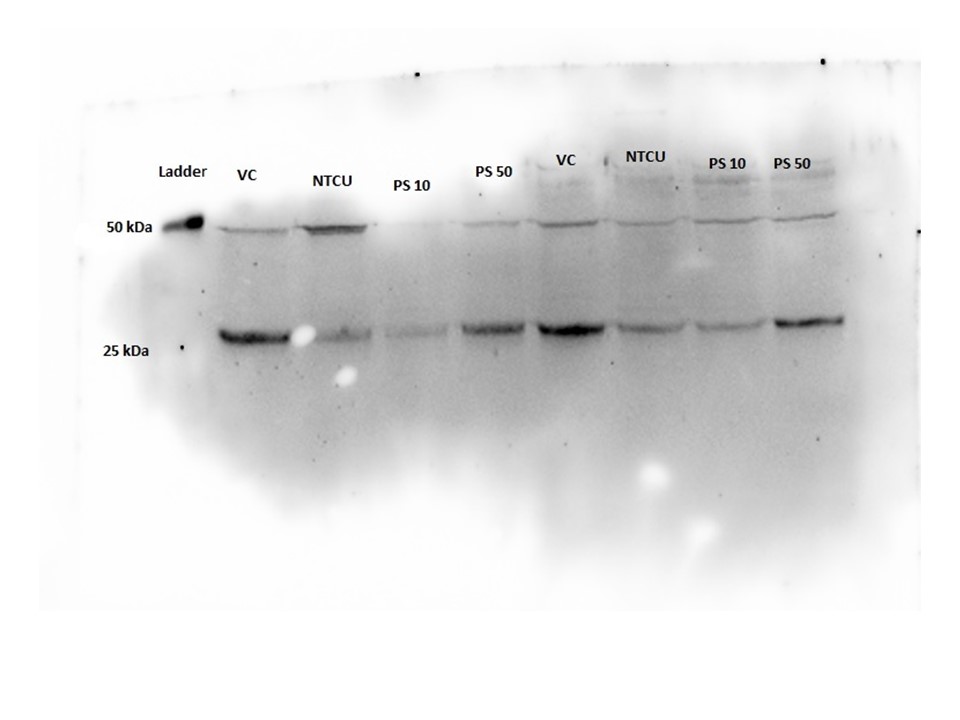


**Supplement Figure 4:** The full blot of the p27 protein band that is included in Figure 3 of the main manuscript. VC: vehicles control group; NTCU: cancer control group treated with NTCU; PS 10: 10 mg/kg of pterostilbene treatment with NTCU; and PS 50: 50 mg/kg of pterostilbene treatment with NTCU.


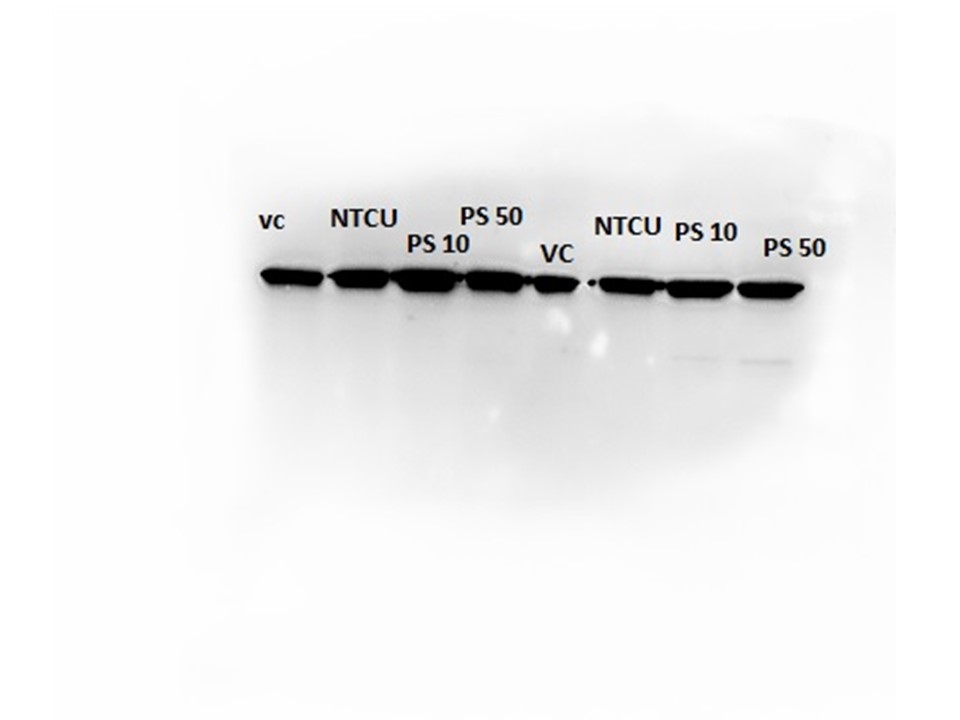


**Supplement Figure 5:** The full blot of the beta-actin protein band that is included in Figure 3 of the main manuscript. Beta-actin acts as a housekeeping protein that was used for the normalization in the quantification of p53and cyclin D1 expression. VC: vehicles control group; NTCU: cancer control group treated with NTCU; PS 10: 10 mg/kg of pterostilbene treatment with NTCU; and PS 50: 50 mg/kg of pterostilbene treatment with NTCU.


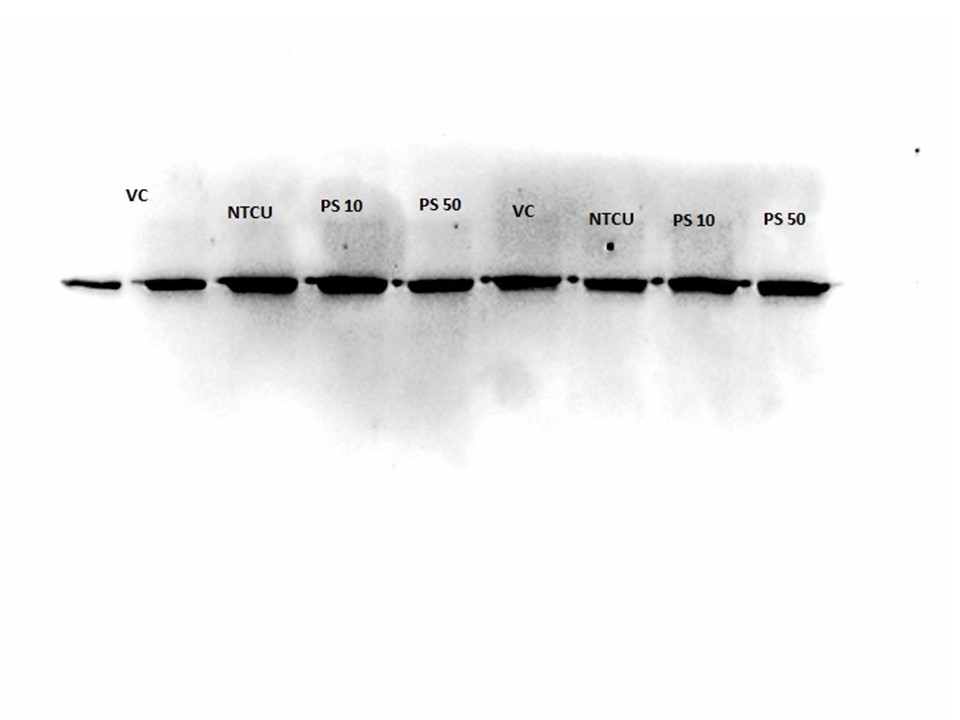


**Supplement Figure 6:** The full blot of the beta-actin protein band that is included in Figure 3 of the main manuscript. Beta-actin acts as a housekeeping protein that was used for the normalization in the quantification of p21and cyclin p27 expression. VC: vehicles control group; NTCU: cancer control group treated with NTCU; PS 10: 10 mg/kg of pterostilbene treatment with NTCU; and PS 50: 50 mg/kg of pterostilbene treatment with NTCU.
